# Using a robotic fish to investigate individual differences in social responsiveness in the guppy

**DOI:** 10.1098/rsos.181026

**Published:** 2018-08-08

**Authors:** David Bierbach, Tim Landgraf, Pawel Romanczuk, Juliane Lukas, Hai Nguyen, Max Wolf, Jens Krause

**Affiliations:** 1 Leibniz-Institute of Freshwater Ecology and Inland Fisheries, Müggelseedamm 310, 12587 Berlin, Germany; 2 Department of Mathematics and Computer Science, Freie Universität Berlin, Institute for Computer Science, Arnimallee 7, 14195 Berlin, Germany; 3 Faculty of Life Sciences, Humboldt University of Berlin, Thaer Institute, Hinter d. Reinhardtstr. 8-18, Berlin, Germany; 4 Department of Biology, Institute for Theoretical Biology, Humboldt Universität zu Berlin, Philippstr. 13, 10115 Berlin, Germany; 5 Bernstein Center for Computational Neuroscience, Humboldt Universität zu Berlin, Philippstr. 13, 10115 Berlin, Germany

**Keywords:** biomimetic robots, fish-inspired robots, *Poecilia reticulata*, robotic fish, social responsiveness

## Abstract

Responding towards the actions of others is one of the most important behavioural traits whenever animals of the same species interact. Mutual influences among interacting individuals may modulate the social responsiveness seen and thus make it often difficult to study the level and individual variation in responsiveness. Here, open-loop biomimetic robots that provide standardized, non-interactive social cues can be a useful tool. These robots are not affected by the live animal's actions but are assumed to still represent valuable and biologically relevant social cues. As this assumption is crucial for the use of biomimetic robots in behavioural studies, we hypothesized (i) that meaningful social interactions can be assumed if live animals maintain individual differences in responsiveness when interacting with both a biomimetic robot and a live partner. Furthermore, to study the level of individual variation in social responsiveness, we hypothesized (ii) that individual differences should be maintained over the course of multiple tests with the robot. We investigated the response of live guppies (*Poecilia reticulata*) when allowed to interact either with a biomimetic open-loop-controlled fish robot—‘Robofish’—or with a live companion. Furthermore, we investigated the responses of live guppies when tested three times with Robofish. We found that responses of live guppies towards Robofish were weaker compared with those of a live companion, most likely as a result of the non-interactive open-loop behaviour of Robofish. Guppies, however, were consistent in their individual responses between a live companion and Robofish, and similar individual differences in response towards Robofish were maintained over repeated testing even though habituation to the test environment was detectable. Biomimetic robots like Robofish are therefore a useful tool for the study of social responsiveness in guppies and possibly other small fish species.

## 1. Introduction

Synchronized behaviours such as collective movements depend on the capability of involved subjects to respond to the actions of their social partners [1–6]. Individual differences in behaviour appear to be a common feature in the animal kingdom [7], and differences in response to social cues have also been repeatedly shown [8–11]. While there is some discussion regarding terminology (see [12]), assessing any response of an individual towards its social environment inevitably requires the presentation of social cues from conspecifics. The use of live conspecifics for this purpose is typically problematic as they often respond themselves to the focal individuals and thereby introduce confounding variation into the experimental design (e.g. [13–16]). Thus, experimenters tried to control for or standardize the potential mutual interactions among subjects, either through training of stimuli subjects [17,18], experimental restriction of interaction possibilities [14,19,20], or by the use of video playbacks [21] or computer animations ([22,23], including virtual realities [24]). Here, we used another technological advancement that might provide a useful tool: biomimetic robots that mimic the appearance and behaviour of live animals and could thus be integrated into groups of live animals [25,26].

Especially fish behaviour has been investigated with biomimetic robots [27–29]. These robots consist of fish-like replicas that move either self-propelled [30–32] or dragged by an external vehicle or manipulator [29,33–46]. Self-propelled robots are often large as all necessary technical equipment has to fit into the robot's chassis and thus can be used mostly for interactions with larger animal species [30,39,47]. Externally dragged and steered robots can be much smaller because most technical equipment is peripheral and thus can be used to investigate smaller species [27].

Recently, sticklebacks have been found to differ consistently from each other in their attraction towards a replica school that is dragged by an overhead wheel and runs at a constant speed [48], a technique that has been also used previously to investigate shoaling tendencies in blind cave tetras (*Astyanax mexicanus*, [49]). These rather stationary presented replicas which allowed researchers to study how animals are attracted to conspecific-like replicas are now complemented with more or less freely moving robots (both self-propelled and externally dragged). These robots enable researchers to go beyond simple scores like measuring the time spent by a focal live animal near an artificial stimulus. With them, it is possible to present live animals with almost the same stimulus they would experience when moving around with live conspecifics. For example, natural swimming behaviour or movement patterns can be presented and robots thus provide the experimental set-ups for comparing the reaction of live animals towards either other live animals or robots.

Biomimetic robots can be either interactive (closed-loop behaviour), which means that they change their behaviour in response to the actions of live animals, or static (open-loop behaviour), which means that they move and behave in predefined, non-interactive ways [25–27]. Biomimetic robots thus provide the experimenter with a diverse toolset to study social interactions such as the ability to provide completely standardized social cues (e.g. through the use of non-interactive open-loop robots, see [33,38,47]). Furthermore, the robot's parameters can be set to either resemble those of focal live individuals or show a sharp contrast with them [41,50,51]. On top, closed-loop-controlled robots allow us to create interactive scenarios that nevertheless follow controlled rules that can be adapted intentionally [36,44,52–55].

One major issue that all artificial social stimuli including biomimetic robots have in common is that experimenters do not know whether responses towards and interactions with them mirror real social interactions or some sort of neophilic explorative behaviour [22,56]. In the current study, we thus asked whether interactions with open-loop biomimetic robots are depicting the tendencies of live animals to respond to social cues (termed hereafter ‘social responsiveness’). If so, we predicted that differences among individuals in their social responsiveness towards a live social partner are maintained, at least in part, in the interaction with the robot. In addition, we predicted that these individual differences in responsiveness towards a biomimetic robot should be consistent over time, e.g. maintained when the same individual is measured multiple times with the biomimetic robot.

To test these fundamental predictions, we used a biomimetic robot (hereafter called ‘Robofish’) that is open-loop controlled and thought to be accepted as a conspecific by live Trinidadian guppies (*Poecilia reticulata*; see [36]). In a first experiment, we explored our first prediction and tested whether among-individual differences in the responsiveness of live guppies towards Robofish are maintained if the same live individual interacts subsequently also with a live social partner. In a second experiment, we explored our second prediction and tested whether among-individual differences in responsiveness towards Robofish are consistent when the same individual is measured several times with Robofish.

## 2. Material and methods

### 2.1. Study organism and maintenance

We used wild-type guppies (*P. reticulata*) for our experiments that have been bred in the laboratory for several generations and originated from wild-caught individuals caught in the Arima River in Trinidad in 2010. Test fish came from large, randomly outbred single-species stocks maintained at the animal care facilities at the Faculty of Life Sciences, Humboldt University of Berlin. We provided a natural 12 L : 12 D regime and maintained water temperature at 26°C. Fish were fed twice daily ad libitum with commercially available flake food (TetraMin™) and once a week with frozen *Artemia* shrimps.

### 2.2. The Robofish system

**Figure 1.**
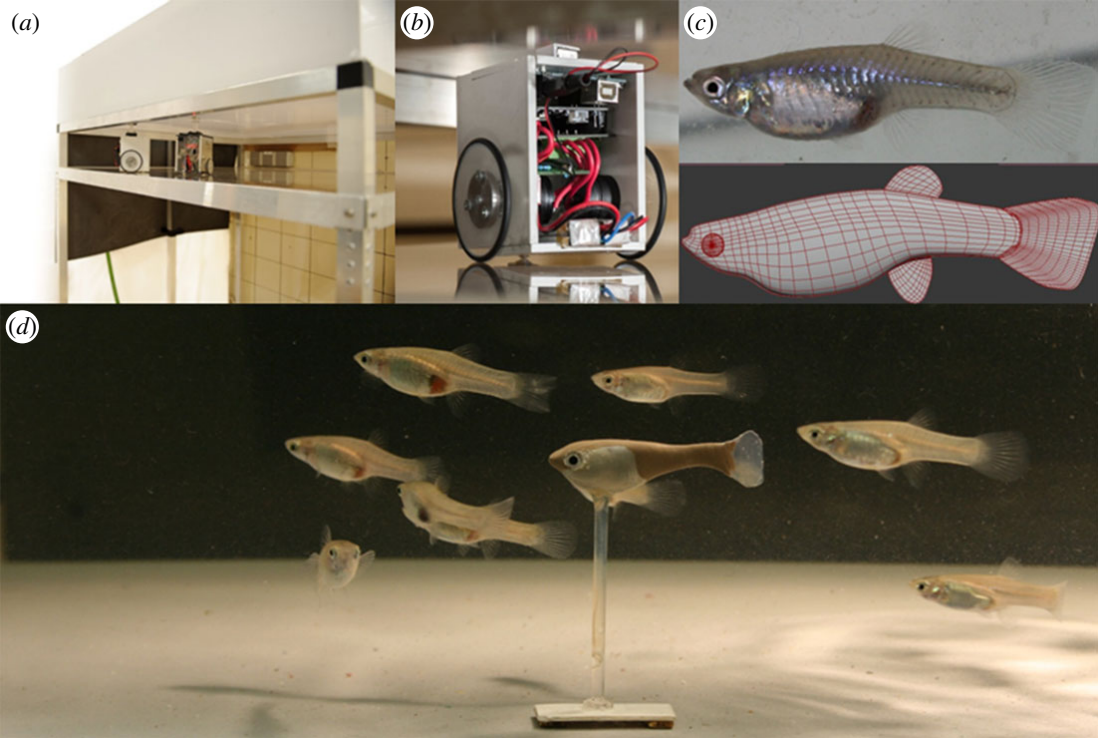
The Robofish system. (*a*) The robot unit is driving on a second level below the test arena. (*b*) Close-up of the robot unit. (*c*) A picture of a live guppy female served as template for the virtual three-dimensional mesh that was printed on a three-dimensional printer. (*d*) Guppy replica with a group of female guppies in the test arena.

The Robofish system consists of a glass tank (88 × 88 cm), which is mounted onto an aluminium rack. A two-wheeled robot can move freely on a transparent platform below the tank (figure 1*a*,*b*). The robot carries a magnet, coupling its motion with a second magnet in the tank above. The second magnet serves as the base for a three-dimensional-printed fish replica (standard length (SL) = 30.0 mm; resembling a guppy female, figure 1*c*). These kind of replicas are readily followed by live guppies (and other fishes), and key features that they reacted to were the glass eyes and natural swimming patterns [33,36]. The entire system is enclosed in a black, opaque canvas to minimize exposure to external disturbances. The tank is illuminated from above with artificial light reproducing the daylight spectrum with a light intensity of 3000 lux at tank level. On the floor, a camera is facing upwards to track the robot. A second camera is fixed above the tank to track both live fish and the robot. Two computers are used for system operation: one PC tracks the robot, computes and sends motion commands to the unit over a wireless channel; the second PC records the video feed of the ceiling camera, which is subsequently tracked by a custom-made software [57]. See the electronic supplementary material, S1 for more details on the robot construction and features.

### 2.3. Experiment 1

#### 2.3.1. Experimental set-up

To compare responses of live focal fish between tests with Robofish and with a live partner, each focal fish was tested once with Robofish and subsequently another time with a live model individual. This was done by testing one half (*n* = 15) of the focal fish first with Robofish and after 2 days with a live model fish, while the other half (*n* = 15) of the focal fish were first tested with a live model fish and after 2 days with Robofish. Focal fish were randomly assigned to start with the Robofish or live model treatment.

#### 2.3.2. Testing responsiveness towards Robofish and live partners

At the beginning of our experiment, we randomly selected adult fish from our stock tanks and marked them individually with visible implant elastomer (VIE) colour tags (see [58]). We used only female guppies in this experiment (in contrast to experiment 2 where both sexes were used, see below) as including males would lead to sexual behaviour being expressed in live fish pairs and possibly influence social interactions [59,60]. After the tagging procedure, we measured body length as standard length (from the tip of snout to the end of caudal peduncle) to the nearest millimetre (focal fish: SL ± s.e.m. = 30.1 ± 0.4 mm, *n* = 30; live model fish: 30.5 ± 0.3 mm, *n* = 30). We are aware that VIE tags might influence social attraction among zebrafish (*Danio rerio*) [61], but this would not systematically bias our results as both focal and model fish were tagged and similar tagging has been used without confounding effects in previous guppy research ([58,62–65], see also [66] for a lack of influence of VIE in cichlid mate choice experiments). However, using VIE tags enabled us to keep guppies in their familiar social group during the whole period of experimentation. Also, when tested a second time, we were still able to identify individuals that were housed in their stock tanks. Furthermore, focal fish can be tested with another live fish without being at risk of losing its identification (ID).

**Figure 2.**
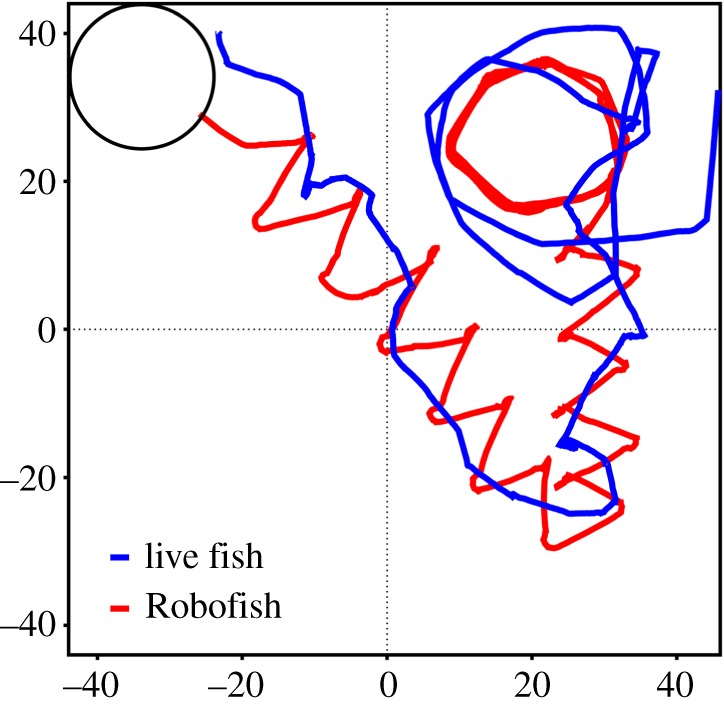
Example track of Robofish with live guppy in an 88 cm × 88 cm test arena. After the live fish left the start cylinder (upper left), Robofish moved in a natural stop-and-go pattern along a zigzagged path to the opposite corner. Upon arrival, Robofish moved to either the bottom left or the top right corner (here: top right) and described a circular path.

To initiate a Robofish trial, we transferred each focal fish into a Plexiglas cylinder located at the upper left corner of the arena (figure 2). The Robofish was also located within the cylinder. After a habituation period of 2 min, Robofish and live fish were released by lifting the cylinder with an automatic pulley system. When the live fish left the cylinder (one body length away from the cylinder's border), Robofish started swimming in a natural stop-and-go pattern [36,67] along a zigzag path to the opposite corner (figure 2). After reaching this corner, the Robofish randomly swam to either the bottom left or the top right corner in which it ultimately described a circular path for three rounds (figure 2). The trial was then terminated and the test fish was transferred back to its holding tank. Each trial was videotaped for subsequent analysis.

Tests with a live social partner were initiated by transferring the focal fish into the start cylinder accompanied by a live companion comparable in size to the Robofish (see above). Again, we lifted the cylinder after 2 min of habituation and videotaped the trial for 2 min, starting when the last fish left the cylinder. Trials involving only live fish were comparable in duration to Robofish trials (live–live: 120 s; Robofish: 124.1 ± 1.9 s; mean ± s.e.m., variation in duration is owing to the stop-and-go swimming pattern of Robofish). After 2 days, the test was repeated; however, those fish that were initially tested with Robofish were then tested with a live companion and vice versa. To further control for our testing procedure, we performed Robofish and live fish trials in an alternating order at each experimental day.

All video recordings were analysed with the custom-made software BioTracker [57] to extract the position and orientation of both interaction partners over time. The video recording frame rate was 30 fps and position tracking (and subsequent analyses of velocities and distances) was done at 5 fps. Based on the tracked positions, we calculated several measures that characterize social interactions and are described in the following section.

#### 2.3.3. Measures of responsiveness

As a simple proxy for the social interaction among subjects, we calculated the inter-individual distance (IID) between focal fish and companion (Robofish or live fish, body centroids) for each trial [2]. It is strongly correlated with other distance-related measures, such as the time fish spent within a specific range (not shown).

As our major goal was to determine a focal individual's responsiveness towards its companions (Robofish or live model), we calculated subject-specific interaction measures for each individual (focal, live model as well as Robofish) within a pair. Freely interacting live fish respond rapidly to the movements of conspecifics by adjusting their own movement patterns [67–72]. To quantify this response in movement patterns, we calculated time-lagged cross-correlations of velocity vectors (TLXC), which allowed us to distinguish how strongly the subjects adjust their own directional velocity towards that of the partner [73]. For any given time lag *τ*, TLXC indicates the strength of the correlation between the velocity vector of the focal individual at time *t* + *τ* and the other companion individual at time *t*. A large positive value implies that on average, the focal individual's directional velocity is similar to that of its companion, whereas values close to zero correspond to a random response and negative values indicate directional velocities in the opposite direction. All first extrema in the cross-correlation can be found for lags less than 6 s. We thus restricted our analysis to lag-times up to *τ* = 6 s. We calculated the cross-correlation averaged over the entire time lag window for both subjects within a pair. Subject-specific TLXCs were then used to calculate a global correlation measure as the difference between the focal fish's average cross-correlation and companion's average cross-correlation (ΔTLXC; positive values: focal fish followed on average; negative values: focal fish led on average). See the electronic supplementary material, S2 for more details on the calculation of TLXC.

#### 2.3.4. Statistical analysis

To see whether the magnitude of social interactions between live pairs and Robofish pairs differed on average, we compared inter-individual distance (log-transformed) and velocity cross-correlations (average TLXCs of subjects in a pair as well as ΔTLXC) between live pairs and Robofish pairs using paired *t*-tests. As one half of the focal fish experienced Robofish first and the other half a live companion, we compared mean distance (log) and ΔTLXC of the Robofish first subset with the live companion first subset but could not find significant differences (unpaired *t*-tests, *p* > 0.08 in all cases).

To investigate differences in interaction patterns between subjects in Robofish and live fish pairs, we compared TLXC between subjects within Robofish and live fish pairs using paired *t*-tests. In addition, we assessed the relationship between IID and TLXC and report Pearson's correlations separate for Robofish and live fish pairs.

Our first main prediction (i) was that among-individual differences in responsiveness towards a live social partner should also be maintained when the same live fish are tested with a Robofish partner. We thus used univariate linear mixed models (LMMs) with IID and TLXC (subject-specific and ΔTLXC) as dependent variables and included focal fish ID as a random factor to calculate the behavioural ‘repeatability’ [74]. The repeatability of a behaviour is defined as the proportion of the total behavioural variance (sum of variation that is attributable to differences among individuals plus variation within individuals) towards the amount of variation that is attributable to differences among individuals. As variance estimates are inherently tied to the total variation present in the response variable, we first mean-centred and scaled the variance of our response variables to 1 within each treatment (e.g. *z*-transformation). No fixed factors were included in the LMM to obtain conservative measures of among- and within-individual variation [74]. A significant repeatability estimate is interpreted as evidence of individual differences that are consistent across both test situations (with live partner and with Robofish). We tested for significance of repeatability estimates using likelihood ratio tests (see [75]).

### 2.4. Experiment 2

#### 2.4.1. Experimental set-up

The aim of our second experiment was to test whether live fish showed consistent differences in their response to Robofish (same robot used as in experiment 1) when tested multiple times with Robofish. As males could not specifically influence Robofish's behaviour (in contrast to females in live pairs during experiment 1), we included also males in this experiment which further helped us to investigate sex differences in the social responsiveness towards Robofish. To do so, male (*n* = 17, SL = 19.5 ± 0.4 mm s.e.m.) and female guppies (*n* = 25, SL = 27.6 ± 0.6 mm) were VIE tagged as described for experiment 1 and kept in 100 l tanks. After one week of acclimatization, all fish were tested three times (once per day with 1 day off between tests) for their responses towards Robofish.

To initiate a trial, focal fish were randomly taken from the stock tank and introduced into an opaque plastic cylinder with a small opening. The opening was closed with a sponge and fish were given 1 min for habituation before the sponge was removed. Robofish was positioned close to the opening at the outside of the cylinder so that the live fish could not see Robofish from the inside but could not miss it once it left the cylinder. Once the focal fish had left the cylinder, Robofish initiated the same zigzag sequence as described for experiment 1. However, this time Robofish did not move in a circular path, but was removed immediately after reaching one corner. A video recording following this protocol is available in the electronic supplementary material, video S1. Video analysis and parameter calculation followed the description provided for experiment 1.

#### 2.4.2. Statistical analysis

To quantify how repeated testing or sex and body size of the fish affected average response towards Robofish, we analysed (log-transformed) IID and ΔTLXC as dependent variables in two separate LMMs with trial (three repeated test runs) and sex as fixed factors and focal fish's body size (SL) as a covariate. Focal ID was included as a random factor to account for repeated tests. Initially, we also included the interaction term ‘sex by body size’, but removed it as it had no significant effect in any model (not shown). As for experiment 1, we furthermore tested for differences in TLXC of focal fish and Robofish using paired *t*-tests and assessed the relationship between IID and TLXC through Pearson's correlations separate for focal fish and Robofish.

Our second main prediction (ii) was that focal fish should maintain individual differences in responsiveness towards Robofish over the three test trials. We used another set of LMMs with IID, subject-specific TLXC and ΔTLXC as dependent variables and focal ID as a random factor. Similar to the analysis described for our first experiment, we first mean-centred and scaled the variance of our response variables to 1 within each trial (e.g. *z*-transformation).

## 3. Results

### 3.1. Experiment 1

#### 3.1.1. Focal fish's average response towards Robofish and live companions

**Figure 3.**
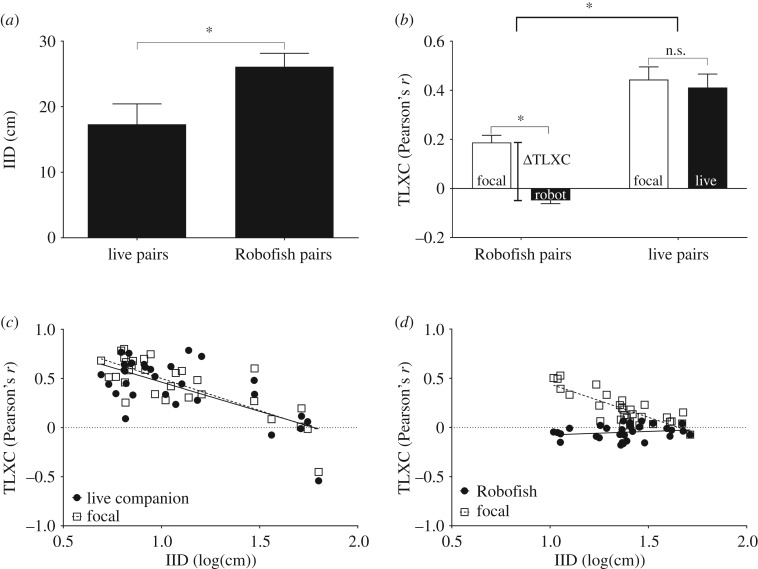
Differences between Robofish pairs and live fish pairs (experiment 1, main text) in (*a*) IID, (*b*) TLXC as well as their relations in (*c*) live fish pairs and (*d*) Robofish pairs. Shown are means ± s.e.m. (*a*,*b*). Asterisks indicate significant differences in *t*-tests (see the main text).

On average, distance between subjects was significantly greater (paired *t*-test, IID: *t*_29_ = −2.353; *p* = 0.022; figure 3*a*) and velocity vector correlations were significantly weaker (average TLXC of both subjects: *t*_29_ = −3.434; *p* = 0.002; figure 3*b*) when focal fish were paired with Robofish compared with trials where the same focal fish were accompanied by live companions. Robofish's velocity vectors were not correlated with those of live focal fish as indicated by velocity vector cross-correlations (TLXC) of Robofish around zero that were significantly weaker than those of the focal fish in Robofish pairs (*t*_29_ = −6.613; *p* < 0.001; figure 3*b*). In live pairs, both fish adjusted their velocities towards each other as indicated by high TLXCs that did not differ between subjects (*t*_29_ = −0.901; *p* = 0.375; figure 3*b*). As a result, ΔTLXC was significantly higher in Robofish pairs compared with live fish pairs (*t*_29_ = −4.031; *p* < 0.001; figure 3*b*).

Velocity vectors of live focal fish were more strongly correlated with their respective partners (both live companions and Robofish) when in close range and correlations decreased when distance among subjects increased (figure 3*c,d*). This was indicated by a significantly negative correlation between IID and focal fish's velocity vector cross-correlations (TLXC) both in Robofish pairs (focal fish: *r*_pearson_ = −0.82; *n* = 30, *p* < 0.001; figure 3*d*) and in live fish pairs (focal: *r*_pearson_ = −0.79; *n* = 30, *p* < 0.001; figure 3*c*). Regarding the companions, live model fish responded with a similar adjustment of their own velocity vectors towards the focal fish and cross-correlations decreased similarly with increasing distance among subjects (companion: *r*_pearson_ = −0.68; *n* = 30, *p* < 0.001; figure 3*c*). However, the non-interactive Robofish did not adjust its movement towards the focal fish at any distance (no correlation detectable for Robofish's TLXC; *r*_pearson_ = 0.2; *n* = 30, *p* = 0.32; figure 3*d*).

In summary, our results indicate that focal fish in Robofish pairs were predominately adjusting their own swimming behaviour to that of Robofish and not vice versa (as intended), while focal fish and live model companions within live pairs were mutually responding towards each other.

#### 3.1.2. Individual differences in social responsiveness

We hypothesized that a focal fish's reaction towards Robofish should reflect its social responsiveness, similar to when tested with a live companion. Although there were general differences in response towards Robofish and a live companion (see above), we found that focal individuals differed consistently across treatments with regard to TLXC and IID (table 1). Only companions' TLXC and ΔTLXC were not repeatable, which is owing to the fact that companions were either Robofish or a live model fish and thus, no systematic consistency can be assumed.

**Table 1.** Behavioural repeatability of subject-specific and pairwise interaction parameters. (Shown are repeatability values obtained from LMMs on treatment-centred and normalized parameters along with 95% credibility intervals (CI) and significance levels from likelihood ratio tests. A significant repeatability indicates consistent individual differences. Significant repeatability values are in italic type face.)

parameter	repeatability	95% CI		*p*-value
experiment 1				
* inter-individual distance (IID)*	*0.44*	*0.35*	*0.54*	*0.007*
* cross-correlation, focal fish (TLXC_focal_)*	*0.40*	*0.29*	*0.51*	*0.015*
** **cross-correlation, companion (TLXC_companion_)	0.00	n.a.	n.a.	0.934
** **difference in cross-correlations (ΔTLXC)	0.09	0.00	0.80	0.767
experiment 2				
* inter-individual distance (IID)*	*0.31*	*0.22*	*0.42*	*0.012*
* cross-correlation, focal fish (TLXC_focal_)*	*0.31*	*0.21*	*0.42*	*0.013*
** **cross-correlation, Robofish (TLXC_Robofish_)	0.10	0.02	0.37	0.320
* difference in cross-correlations (ΔTLXC)*	*0.32*	*0.23*	*0.43*	*0.011*

### 3.2. Experiment 2

#### 3.2.1. Is social responsiveness towards Robofish consistent over repeated testing?

**Figure 4.**
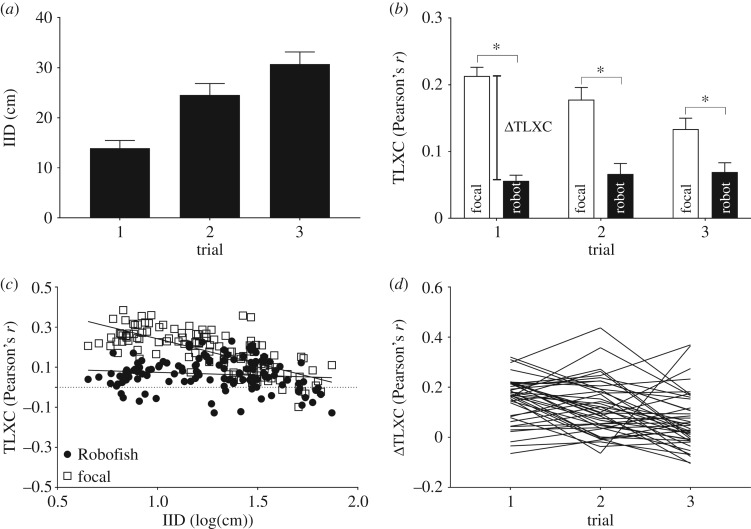
Results from repeated testing with Robofish. (*a*) IID and (*b*) TLXC. Note that IID and ΔTLXC for trial 1 were significantly different from those for trials 2 and 3 (post hoc least significant difference tests). (*c*) Relationship between IID and TLXC separate for Robofish and focal fish. Shown are pooled data from all three trials. (*d*) ΔTLXCs over the repeated testing. Each line represents a focal individual's ΔTLXC in each of the three consecutive trials. Shown are means ± s.e.m., (*a*,*b*). Asterisks indicate significant differences in *t*-tests (see the main text).

We detected a significant reduction in the focal fish's response to Robofish over time (significant effect of factor ‘trial’ in LMM; IID: *F*_2,82_ = 30.908, *p* < 0.001, figure 4*a*; ΔTLXC: *F*_2,82_ = 11.737, *p* < 0.001, figure 4*b*). Neither body length of the test fish nor sex had a significant effect in either model (not shown). As found in experiment 1, the levels of ΔTLXC were owing to the high levels of TLXC of the focal individuals that were significantly stronger than that of Robofish which were again around zero (figure 4*b*). This indicates that focal fish but not Robofish adjusted their directional velocities to their social partner. Also, focal fish's but not Robofish's TLXC were significantly negatively correlated with IID (focal fish: *r*_pearson_ = −0.75; *n* = 126, *p* < 0.001; Robofish: *r*_pearson_ = −0.16; *n* = 126, *p* = 0.063; figure 4*c*). This pattern is similar to Robofish pairs in experiment 1 and shows that focal fish adjusted their directional velocities towards Robofish more strongly when in close range.

Despite the response reduction, we found focal fish to largely maintain their individual differences in responsiveness when interacting with Robofish over the course of the repeated tests as indicated by significant repeatability estimates for IID, TLXCs and ΔTLXC (table 1 and figure 4*d*).

## 4. Discussion

The aim of our study was to provide evidence that live fish's interactions with our biomimetic Robofish represent biological meaningful social interactions. Although responses towards Robofish were weaker (compared with a live companion), our results showed that guppies were consistent in their individual responses between a live companion and Robofish (first experiment). As predicted, individual differences in response towards Robofish were also maintained over repeated testing with Robofish even though a reduction in response was detectable (second experiment).

Although guppies readily followed the moving Robofish, guppies in pairs of only live fish were on average significantly closer to each other and had significantly stronger correlated velocity vectors (TLXC) than guppies tested with Robofish. Robofish's zero values in the velocity vector correlations suggest that these overall weaker responses were owing to the Robofish's inability to adjust its behaviour towards the live partner fish (non-interactive, open-loop behaviour). Despite Robofish's non-interactive behaviour, focal fish showed similar distance-dependent patterns in their adjustment of their own velocity vectors towards Robofish as they showed when interacting with a live social partner, e.g. stronger velocity vector correlations were found at closer ranges in both test situations. Weaker responses of live fish towards open-loop (e.g. non-interactively controlled) compared with closed-loop robots are well known from other studies [36,43,76] and should therefore be viewed as a systematic characteristic of tests with open-loop biomimetic robots at least in studies involving fishes (see also [27]). Furthermore, biomimetic robots are yet not able to reproduce exactly the same cues as live conspecifics but can reproduce certain ‘social releaser’ cues [77] that make them become an accepted social interaction partner for live animals (see also discussion in [36]). Non-interactive open-loop robots provide a unique tool for the study of individual differences in social responsiveness (and other social behaviour, for example courtship [38] or aggressive interactions [34,35]) as they allow testing of all individuals from a sample with a similar (almost identical) set of social stimuli. Observed among-individual differences in response are then inevitably caused by focal fish's ID and not by mutual interactions between focal and stimuli animals that are often encountered in tests with live social stimuli [69,78].

As predicted, we found consistent individual differences in responsiveness both at pair level (e.g. in IIDs) and in focal fish's tendency to adjust its velocity vectors to that of its social partners across both test situations (i.e. with a live companion and Robofish, first experiment) as well as when repeatedly tested with Robofish (second experiment). We detected an average decrease in response towards Robofish over the course of the three repeated tests. This is a common feature when individuals are tested multiple times in the same context regardless of whether live fish or robots are used because animals habituate to the test tank [7].

Our initial predictions could be confirmed and we are therefore confident that reactions towards Robofish provide a consistent and reliable measure for social responsiveness in live guppies. This conclusion is in line with other studies that used dragged robots to attract live guppies [34] as well as studies on other fish species [27]. For example, similar positive validation efforts have been made for zebrafish's (*D. rerio*) responses towards biomimetic robots [47,50,51,79,80]. Experiments with sticklebacks and circulating robot shoals further suggest that even robots with a more or less stationary movement pattern are able to be accepted as conspecifics and elicit individual differences in responsiveness [48].

An alternative explanation for our results could be seen in the fact that animals are known to differ in their tendency to explore new objects [8], and Robofish might just be perceived as such a new object rather than a conspecific. Previous recommendations for the validation of synthetic (artificial) stimuli argue that meaningful biological reactions in animals can be assumed if reactions towards an artificial stimulus, at least in part, mirror the reaction that is observable in live animals put into similar contexts [22]. This was the case for Robofish as live guppies showed similar patterns of distance-dependent adjustment in their velocity vectors when tested with Robofish and live partners. However, the response reduction found in the second experiment could, in part, be owing to such ‘exploratory curiosity’ of the live fish. Even if we cannot rule out that guppies, in part, are attracted to Robofish through a ‘exploratory curiosity’ mechanism, the fact that live fish maintained their individual differences in social responsiveness when interacting with a live partner also when interacting with Robofish clearly validates our open-loop Robofish as a tool for the study of social responsiveness.

Investigating social interactions of live animals most often relies on the observation of animal groups with only little room for directly manipulating individual members of the group. While several new methods have been developed to provide animals with controllable artificial social stimuli [22,56], only movable biomimetic robots allow investigators to manipulate social cues within moving animal groups. Integrating such biomimetic robots into groups of live animals is a crucial approach to get meaningful insights into social interactions. Our study shows that live guppies react in a weaker but similar way to a non-interactive biomimetic robot—the Robofish and, most importantly, maintained individual differences shown in the interaction with a live social partner also when interacting with Robofish. Through its highly standardized behaviour, Robofish is thus a useful tool to investigate individual differences in social responsiveness in live guppies and possible other teleost fishes.

## Supplementary Material

Text S1: Details on Robofish design and features

## Supplementary Material

Text S2: Calculation of average time-delayed cross-correlation (TLXC)

## Data accessibility

Data can be accessed via Dryad: http://dx.doi.org/10.5061/dryad.4823jr1 [81].
