# Supplementary material for: Using a robotic fish to investigate individual differences in social responsiveness in the guppy
Source: R Soc Open Sci. 2018 Aug 8;5(8):181026. doi: 10.1098/rsos.181026 (PMC6124066; doi:10.1098/rsos.181026)
Supplement: Text S1: Details on Robofish design and features [file rsos181026supp1.pdf]

Electronic Supplemental Material S1

Using a robotic fish to investigate individual differences in social responsiveness in the guppy (S1)

David Bierbach*, Tim Landgraf*, Pawel Romanczuk, Juliane Lukas, Hai Nguyen, Max Wolf, Jens Krause

** Authors contributed equally*

Robot design

The two-wheeled robot is a cuboid with dimensions 7×7×10 cm built from aluminum and plastic plates. It houses one Arduino-compatible board for main processing and WiFi communication (ESP-12F). Custom-built wheels (diameter = 6 cm) with rubber gasket rings are directly connected to two dc gear motors (2622S006B SC 33:1, Dr Fritz Faulhaber GmbH & Co. KG) which include a speed controller for pulse width modulation (PWM) control. These controllers allow for precise speed actuation. The main power is supplied by a LiPo battery pack (two-cell, 7.4 V nominal output, Conrad Electronic SE). The motors are driven via PWM signals generated by the ESP-12F. A voltage divider scales down the raw battery output, which then can be measured on an analog pin of the ESP board with respect to an internal reference. The robot carries a neodymium magnet at the tip of a plastic rod held up against the glass tank maintaining an air gap. The tank itself is made of glass, which due to the mass of the water column bends slightly downwards. The distance between the magnet and the tank therefore varies from less than 1 mm near the center of the tank to approximately 2 mm in the periphery. The magnets' poles are aligned in parallel to the motion plane and the replica's base aligns with the robot. This steady coupling allows on-the-spot rotations and fast accelerations of the replica.

Robot control and motion models

The control software sends motion commands at a frequency of 25 Hz, as determined by the bottom camera's frame rate. In each time step, a command packet is issued and sent to the individual robot via WiFi (UDP). Each robot has a unique IP-address and only receives its respective packets. We use a fixed length protocol with a 2 bytes header and 12 bytes data.

The motion control is organized in layers:
(1) Arbitrary behavior layer that generates global target points. These behaviors are required to implement a simple interface and are loaded as shared objects.
(2) Trajectory manipulation layer, which converts high level control commands into a command queue. Here motion types and navigation automation are done.
(3) The ego-motions layer, which converts the next command from the queue into motor speeds using a p/d/i controller with overlay speed-controlling. When operating on a command sequence, the ego-motions layer (3) will check for every camera frame whether the current low-level target point has been reached (with a tolerance of 3 cm). If so, it pops this command from the queue. Thus, departures from the hypothetical trajectory are instantaneously corrected and errors are low (see below).

The motors are able to propel the system to a maximum forward and turning velocity of approximately 60 cm/s and 860 deg/s, respectively. The minimum velocities are approximately 1 cm–s and 15 deg–s.

No odometric feedback control has been used. The robots' motion is solely controlled via vision feedback as to avoid drift. Most of the computation is outsourced to personal computers. Currently, the robot's firmware implements the following: (1) Sending status packets back to the control computer at 1 Hz, including the robot's unique identifier and battery level. (2) It generates PWM signals according to the received motor speed values. (3) It can be configured to run in jump-start mode. To overcome initial friction and inertia, this mode prepends higher PWM duty cycles when motor speeds are low.

Fish Tracking

The tracking algorithm and software presented in [2] have been ported into a plugin for the BioTracker [3] framework with minor changes to the algorithm itself. Interactions of robots and live fish are observed from above the tank by a second camera. In order to detect all individuals, we use a background subtraction procedure that models foreground and background pixel distributions as a mixture of Gaussians [4]. Since the tank's bottom and walls are laminated with white plastic, the fish appear as clear dark objects in the video images. Once converged, the background model shows an empty tank such that the difference image of background and current live frame exhibits distinct positive peaks. The individual fish (either Robofish or live fish) are detected by first applying a global threshold to the difference image. All regions having above-threshold values are then treated similarly to the blobs in the robot tracking. Additionally, the blobs are assigned an orientation by integrating the motion vector over a fixed time window, when fast movements are detected. The system assigns an ID to every fish blob and tracks it using an ellipsoid model.

Replica construction and performance comparison

The fish replicas were 3D-printed standard models (see [5, 6] for a similar approach). The 3D mesh was produced manually from several template photographs. The printed replicas were then painted and finished and equipped with glass eyes (3mm diameter, model: kristall B11; Lauschaer Glasaugen, Frank Weigelt). Replicas resemble female guppies as previous research showed that females were similarly preferred as shoaling partners by both sexes while males are often avoided by females due to the typically high rates of male sexual harassment (see [7]). We used one replica with a standard length (snout to caudal peduncle) of 30 mm.
For the current experiments, Robofish moved along a zig-zag pattern with a maximum speed of 35 cm/s (Video S1). The robot further exhibited a stop-and-go pattern where it accelerated and subsequent decelerated to almost 0 cm/s while afterwards accelerating again to the predefined maximum speed. This stop-and-go motion pattern leads to an average speed of 4.15 cm/s (± 0.27 SD). In order to test how accurate Robofish swam along the predefined zigzag path, we measured the deviation (in cm) of the tracked trajectories from the hypothetical trajectory at 5 locations along the track for our data from experiment 1 (N=30 Robofish tracks, 150 data points). Robofish's deviation from the hypothetical trajectory followed a Gaussian distribution (D'Agostino and Pearson normality test; $K2 = 0.11$; $P = 0.95$) with a mean of 0.02 cm (lower 95%CI = -0.14; upper 95%CI = 0.19). This means that 90% of the analyzed data points were within a range of -1.3 cm and 1.4 cm around the hypothetical trajectory.

References

1. Landgraf T., Nguyen H., Schröer J., Szengel A., Clément R.G., Bierbach D., Krause J. 2014 Blending in with the shoal: Robotic fish swarms for investigating strategies of group formation in guppies. In *Biomimetic and Biohybrid Systems* (eds. Duff A., Lepora N., Mura A., Prescott T., Verschure P.M.J.), pp. 178-189, Springer International Publishing.
2. Landgraf T., Bierbach D., Nguyen H., Muggelberg N., Romanczuk P., Krause J. 2016 RoboFish: increased acceptance of interactive robotic fish with realistic eyes and natural motion patterns by live Trinidadian guppies. *Bioinspir Biomim* **11**(1), 015001.
3. Mönck H.J., Jörg A., Falkenhausen T.v., Tanke J., Wild B., Dormagen D., Piotrowski J., Winklmayr C., Bierbach D., Landgraf T. 2018 BioTracker: An open-source computer vision framework for visual animal tracking. *arXiv:180307985*.
4. Zivkovic Z., Heijden F.v.d. 2004 Recursive unsupervised learning of finite mixture models. *IEEE Transactions on Pattern Analysis and Machine Intelligence* **26**(5), 651-656. (doi:10.1109/TPAMI.2004.1273970).
5. Ruberto T., Polverino G., Porfiri M. 2017 How different is a 3D-printed replica from a conspecific in the eyes of a zebrafish? *Journal of the Experimental Analysis of Behavior*, n/a-n/a. (doi:10.1002/jeab.247).
6. Phamduy P., Polverino G., Fuller R.C., Porfiri M. 2014 Fish and robot dancing together: bluefin killifish females respond differently to the courtship of a robot with varying color morphs. *Bioinspir Biomim* **9**(3), 036021. (doi:10.1088/1748-3182/9/3/036021).
7. Magurran A.E. 2005 *Evolutionary ecology: The Trinidadian guppy* Oxford, Oxford University Press.
