# Supplementary material for: Using a robotic fish to investigate individual differences in social responsiveness in the guppy
Source: R Soc Open Sci. 2018 Aug 8;5(8):181026. doi: 10.1098/rsos.181026 (PMC6124066; doi:10.1098/rsos.181026)
Supplement: Text S2: Calculation of average time-delayed cross-correlation (TLXC) [file rsos181026supp2.pdf]

Electronic Supplemental Material S2

Using a robotic fish to investigate individual differences in social responsiveness in the guppy (S2)

David Bierbach*, Tim Landgraf*, Pawel Romanczuk, Juliane Lukas, Hai Nguyen, Max Wolf, Jens Krause

** Authors contributed equally*

Calculation of average time-delayed cross-correlation (TLXC)

We calculated the time-delayed normalized cross correlation function for different values of the lagtime τ: $C(\tau)=< v(t)v_f(t+\tau)>_t$ with v_f being the velocity of the focal individual and $<...>_t$ indicating a time average over all points where $v(t)v_f(t+\tau)$ is defined. As the cross correlation, requires the shifting of the velocity vector of the focal individual by k points, with $\tau=k\Delta t$ (Δt: time difference between two time frames), the time average for different τ (different k) has to be taken over $k_{avg}= \lfloor (T-\tau)/\Delta t \rfloor$ points where $v(t)v_f(t+\tau)$ is defined. Here T is the total trajectory duration used in experiments $T=120s$. For example for $\tau =3s$ and $\Delta t=0.111$, we obtain k_{avg}= 1053. Eventually, we calculated **TLXC** as the average $C(\tau)$ over a finite range of timelags (τ =0 - 6s): **TLXC**=$< C(\tau) >$.
